# Clinical Management and Gene Mutation Analysis of Children with Congenital Hyperinsulinism in South China

**DOI:** 10.4274/jcrpe.galenos.2019.2019.0046

**Published:** 2019-11-22

**Authors:** Aijing Xu, Jing Cheng, Huiying Sheng, Zhe Wen, Yunting Lin, Zhihong Zhou, Chunhua Zeng, Yongxian Shao, Cuiling Li, Li Liu, Xiuzhen Li

**Affiliations:** 1 Guangzhou Women and Children’s Medical Center, Clinic of Genetics and Endocrinology, Guangzhou, China; 2 Guangzhou Women and Children’s Medical Center, Clinic of Pediatric Surgery, Guangzhou, China; ★ Contributed equally to this work

**Keywords:** Congenital hyperinsulinism, clinical management, gene mutation

## Abstract

**Objective:**

To explore the clinical presentation and molecular genetic characteristics of a cohort of congenital hyperinsulinism (CHI) patients from southern China and also to explore the most appropriate therapeutic approaches.

**Methods:**

We retrospectively reviewed a cohort of 65 children with CHI. Mutational analysis was performed for *KCNJ11* and *ABCC8* genes. The *GLUD1* gene was sequenced in patients with hyperammonaemia. GCK gene sequencing was performed in those patients with no mutation identified in the *ABCC8, KCNJ11* or *GLUD1* genes.

**Results:**

*ABCC8* mutations were identified in 16 (25%) of the cohort, *GLUD1* mutations were identified in five children, and no *KCNJ11* or *GCK* mutations were identified. Moreover, some unique features of *ABCC8* gene mutations in southern Chinese CHI patients were found in this study. The most common mutation was a deletion/insertion mutation p.Thr1042GlnfsX75 was found in five unrelated patients, which possibly represents a relatively common mutation in southern China. Five novel *ABCC8* mutations were detected. The mutations were p.Phe5SerfsX72, p.Gln273ArgfsX85, p.Leu724del, p.Asp1447Gly and IVS 25-1G>T. Five compound heterozygous mutations of *ABCC8* gene were identified in this study, and three of these patients were diazoxide-responsive. Forty patients were diazoxide-responsive, 13 patients were diazoxide-unresponsive and 12 patients received dietary treatment only. A pancreatectomy was performed in 10 patients who were unresponsive to medical treatment.

**Conclusion:**

To the best of our knowledge, this is the first study of CHI in south China. Mutations in ABCC8 are the most common causes of CHI in this cohort. Diazoxide and dietary treatment were effective in most patients. Multicentre studies are necessary to obtain the long-term follow-up characteristics of such patients at a national level.

**What is already known on this topic?**

Congenital hyperinsulinism (CHI) is a rare inherited disease characterized by unregulated insulin secretion and profound hypoglycemia. There are few reports pertaining to patients with CHI from south China.

**What this study adds?**

This is the first study investigating the clinical features, molecular genetic characteristics and treatment, including the optimal therapeutic approach, in patients with CHI in south China.

## Introduction

Congenital hyperinsulinism (CHI) is the most frequent cause of persistent hypoglycaemia in neonates and infants. CHI occurs due to the dysregulated and inappropriate secretion of insulin from pancreatic β cells (1). The incidence of CHI is estimated to be 1 in 40,000-50,000 live births in northern Europe (2,3) and 1 in 2,500 births in Saudi Arabia (4). There are no nationwide data regarding the incidence of this disorder in China.

Inappropriate insulin secretion can suppress the production of ketone bodies, which serve as an alternative fuel during hypoglycemia. The lack of glucose and the deprivation of alternative fuels for the brain will increase the risk of brain damage in these patients (5). To date, mutations in 14 different genes, namely *ABCC8, KCNJ11, GLUD1, GCK, HADH, SLC16A1, UCP2, HNF1A* (6)*, HNF4A *(7)*, HK1 *(8),* PGM1* (9), *PMM2* (10), *CACNA1D *(11), and *FOXA2 *(12), that lead to dysregulated secretion of insulin have been described. The most common forms of CHI are due to defects in *ABCC8* and* KCNJ11*, which encode the ATP-sensitive potassium (KATP) channel subunits of the sulfonylurea receptor (SUR1) protein and inwardly rectify potassium channel (Kir6.2) proteins, respectively (13). Both these genes are located on chromosome 11p15.1 (14).

The clinical presentation can be varied, ranging from completely asymptomatic to mild or severe disease that is unresponsive to medication and requires surgical intervention (15). Diazoxide is the first-line agent for the treatment of CHI. Diazoxide binds to the SUR1 subunits and opens the KATP channel, thereby preventing depolarization of the β-cell membrane and insulin secretion (16). If necessary, those who are unresponsive to medical therapy may undergo surgical treatment. Histologically, CHI is divided into three subgroups. These are the diffuse, focal and atypical forms of CHI. Children with diffuse CHI may require a near-total pancreatectomy which carries the attendant risk of diabetes mellitus and exocrine pancreatic insufficiency, whereas the focal form will only require a limited, focal lesionectomy. Conventional radiological imaging is often used but is unable to distinguish between the two forms (17). ^18^F-DOPA positron emission tomography/computed tomography (PET/CT) scanning is an accurate and non-invasive technique to differentiate focal and diffuse types of CHI (18). Unfortunately, this imaging method is not available in southern mainland China. Genetic analysis may provide important diagnostic clues, particularly in the absence of specific imaging modalities, during the investigation of CHI cases. Children with the diffuse form of CHI due to recessive mutations in *ABCC8* and *KCNJ11* usually do not respond to diazoxide. Focal forms are sporadic and associated with a paternally inherited mutation in *ABCC8/ KCNJ11* genes (19).

Although the clinical characteristics and genetic aetiology of CHI patients have been described in some studies in China (20,21,22), little is known about CHI in southern China. We first reported our experiences with the management of CHI in 12 children in 2009 (23).

The objectives of the current study were to investigate the clinical presentation and molecular genetic characteristics of a group of patients with CHI in southern China and also to explore the most appropriate therapeutic approaches.

## Methods

### Subjects

Enrolled patients included those diagnosed with CHI who were hospitalized in the Guangzhou Women and Children’s Medical Center from November 2012 to June 2017. Most of the patients with CHI came from the Guangdong, Guangxi, Jiangxi, Hunan, Hubei, Yunnan and Hainan provinces of southern China. Serum ammonia levels were checked in all cases. All infants and children were diagnosed with CHI based on clinical and biochemical criteria which were as follows: whether serum insulin was simultaneously detectable (>2 mU/l) concurrent with hypoglycaemia (blood glucose <2.6 mmol/l); along with evidence of elevated glucose requirements (glucose infusion >8 mg/kg/min) in the absence of ketosis or ketonuria; and an inappropriate glycaemic response to glucagon injection at the time of hypoglycaemia (15). Patients with a secondary cause of hypoglycaemia such as perinatal asphyxia, prematurity, intrauterine growth restriction, maternal diabetes and syndromic forms were excluded. Being responsive to diazoxide was defined as maintaining blood glucose above 3.5 mmol/l after a short period of fasting which varied depending on the age of the patient: 4 hours in neonates; 8 hours in infants; and 12 hours in children (6). Clinical data were obtained from medical records. The study was reviewed and approved by the Ethical Committee of Guangzhou Women and Children’s Medical Center (2016022210).

### Genetic Analysis

Genomic DNA was extracted from peripheral blood leucocytes, using a kit according to the manufacturer’s instructions (Qiagen, Hilden, Germany). All exons and intron-exon boundaries of the *ABCC8* and* KCNJ11* genes were amplified by polymerase chain reaction, purified and sequenced. The sequences were analysed and compared to the wild-type published reference sequences (NM_000525 for* KCNJ11* and NM_000352.3 for *ABCC8*) using Chromas software (Technelysium Pty Ltd, South Brisbane, Australia). The* GLUD1* gene was sequenced in patients with hyperammonaemia (HA), whereas *GCK* gene sequencing was subsequently performed in those patients with no mutation identified in the *ABCC8, KCNJ11 *or* GLUD1* genes. The novelty of mutation sites were determined by searching the Human Gene Mutation Database (HGMD, http://www.hgmd.cf.ac.uk) and the National Center for Biotechnology Information database (https://www.ncbi.nlm.nih.gov/clinvar). All variations were identified in this study using the Single Nucleotide Polymorphism database (dbSNP) (http://www.ncbi.nlm.nih.gov/SNP) and the 1000 Genomes Project database (https://www.internationalgenome.org/1000-genomes-project-publications). Intronic variants were analysed with GenSCAN (http://genes.mit.edu/GENSCAN.html). To test the pathogenicity of novel missense mutations, Polymorphism Phenotyping (PolyPhen, http://genetics.bwh.harvard.edu/pph) and Sorting Intolerant from Tolerant (https://sift.bii.a-star.edu.sg/) were used.

### Treatment and Follow-up

Neonatal history, clinical presentation, treatment and complications were analysed in the CHI patients. Intravenous glucose infusion to maintain blood glucose levels of >2.8 mmol/l, nutritional therapy and diazoxide treatment were initiated immediately upon diagnosis. Nutritional therapy included frequent meals enriched with complex carbohydrates and nasogastric feeds at midnight. A glucose polymer, maltodextrin (Malt Extract, Wakodo, Asahi Group Foods, Ltd., Japan), was utilized in infants younger than six months of age. Subjects older than six months were given supplemental uncooked corn starch through a nasogastric tube between meals, before bedtime and for night-time feeds. Diagnostic tests for protein-sensitive hypoglycemia were performed in five patients with HI/HA syndrome. The blood glucose concentrations of all five patients decreased following the protein load and an age-adjusted daily diet consisting of a protein combination with fat and carbohydrate was started.

Diazoxide was started in a dose of 10 mg/kg/day, given in three divided doses. When diazoxide treatment was effective, the dosage was reduced to the effective minimum. Oral hydrochlorothiazide (1-2 mg/kg/day) was used in conjunction with diazoxide to counteract the fluid-retaining properties of diazoxide. All patients treated with diazoxide were carefully monitored for fluid and sodium retention. In three children who were not responsive to diazoxide, octreotide (5-25 µg/kg/day) injections were administered.

Pancreatectomy was implemented in patients not responding to medical therapy. Clinical follow-up was initiated one month after hospital discharge and continued at intervals of three months subsequently. Self-monitored blood glucose levels were recorded. Brain damage was evaluated by a pediatric neurologist at the time of diagnosis and at each three monthly follow-up.

### Statistical Analysis

The results were analysed using the SPSS 17.0 program (IBM Inc., Armonk, NY, USA) and were expressed as the mean±standard error of the mean (mean±SE) and in percentages (%). The Student’s t-test and the Wilcoxon test were used for statistical analysis of the data. All p values less than 0.05 were considered significant.

## Results

A total of 65 patients (47 males and 18 females) with a diagnosis of CHI were included in the study. Age at diagnosis ranged from immediately following birth to seven years old. Twenty-three patients (35.4%) were macrosomic and their mean birth weight was 3,690 g. Sixty-two patients were born at term. The CHI symptoms were first noted during the neonatal period in 29 patients (44.6%), during the infancy period (1-12 months) in 26 patients (40%) and during childhood (>12 months) in 10 patients (15.4%).

Of the 65 patients, 13 were diazoxide-unresponsive, 40 patients were diazoxide-responsive and 12 received dietary treatment only. Patients were divided into two groups based on diazoxide responsiveness; Group 1, diazoxide-unresponsive and Group 2, responsive to diazoxide or dietary treatment. The clinical and biochemical characteristics of the patients in the two groups are presented in Table 1. Age at onset of CHI was significantly different between these two groups. The neonatal form comprised 92.3% of Group 1, but only 32.7% of Group 2. There was a significantly higher incidence of epilepsy in Group 2 than in Group 1 (p<0.05). The time between symptom manifestation and diagnosis ranged from one day to six years, and the duration was again significantly longer in Group 2. A patient (Case 61) in Group 2 was six years old and was initially misdiagnosed as having a seizure disorder before the hypoglycemia was detected.

**Figure 1:**
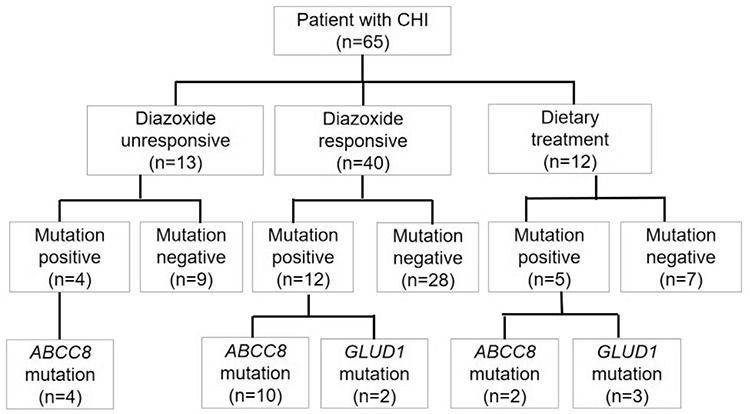
Distribution of patients according to mutation analysis results and treatment choices for patients with congenital hyperinsulinism CHI: congenital hyperinsulinism

In our study, *ABCC8* mutations were identified in 16 children (25% of the cohort), and no *KCNJ11* mutations were identified on KATP channel gene mutation analysis. Five patients with persistent HA had mutations in *GLUD1* (Figure 1). No variants were found in the *GCK* gene. Fifteen different *ABCC8 *mutations were discovered, five mutations were compound heterozygous, 11 were heterozygous and none were homozygous (Table 2). Among the children who carried compound heterozygous mutations, diazoxide treatment was effective in three children. Treatment was not effective in one child and one child was regulated with diet.

The most common mutation was a deletion/insertion mutation c.3224-3226delACC ins CAGCCAGGAACTG (p.Thr1042GlnfsX75) found in five unrelated patients, which possibly represents a relatively common mutation in southern China. Five novel *ABCC8* mutations (p.Phe5SerfsX72, p.Gln273ArgfsX85, p.Leu724del, p.Asp1447Gly and IVS 25-1G>T) were identified. Of the novel mutations, two were frameshift mutations, one was a deletion mutation, one was a missense mutation and one was a splice site mutation. In accordance with the guidelines of the American College of Medical Genetics and Genomics (ACMG) (24), two variants were perceived as “pathogenic” and three variants were predicted as “likely pathogenic”. A novel heterozygous variant in *ABCC8* gene was identified in case 22. The patient has now been on therapy with diazoxide for more than one year at a dose of 5 mg/kg/day with normal growth and development. In one case (patient 59), two novel mutations were identified. This girl was macrosomic at birth. Hypoglycemia was first detected on day three after birth at a local hospital, and improved with frequent feedings. However, her parents did not monitor her blood glucose. She was admitted to our hospital at age 14 months for brief generalized convulsive periods. Laboratory tests revealed hypoglycemia (blood glucose: 2.5 mmol/L) HI (plasma insulin level: 5.9 µIU/mL) when she had an episode. Four hourly daytime feeds (solids and cow’s milk) and four hourly uncooked cornstarch (1.6 g/kg) could maintain the blood glucose above 3.5 mmol/L. During six months follow-up, there was no episodes of hypoglycemia. However, she had sustained hypoglycaemic brain injury with global developmental delay.

In this study, 16 parents underwent genetic analysis. Five patients (patients 1, 5, 10, 14 and 18) had paternally inherited monoallelic mutations. Of the five patients, three were diazoxide-unresponsive and two were diazoxide-responsive. In the three diazoxide-unresponsive patients (patients 1, 5 and 10), diffuse pancreatic disease was confirmed following surgery. One patient (patient 3) had two heterozygous mutations: one missense mutation c.314A>C (p.His105Pro) in exon 3, inherited from his father and a nonsense mutation c.2800C>T (p.Gln934X) in exon 23 inherited from his mother. He was diazoxide-unresponsive, which had been previously reported (25). The ^18^F-DOPA PET/CT scan indicated a focal lesion in the head of the pancreas, whereas the histology of the resected pancreas showed atypical form. The enlargement of pancreatic β-cell nuclei were distributed mainly in the head but included the body and tail of the pancreas. The abnormal active endocrine cells were not restricted to a focal lesion nor were they present throughout the entire pancreas.

The *GLUD1 *gene was detected in patients with hypoglycaemia, HI and mild HA. Three different heterozygous mutations in the *GLUD1* gene were identified in five patients. The p.Arg322 His mutation was found in patients 54, 55 and 56. Patients 55 and 56 were sisters. The mutation was autosomal and dominantly inherited from their father, who was an asymptomatic carrier. The p.Ser498Leu mutation was found in patient 52 and the p.Asn463Asp mutation in patient 53. All mutations have been previously reported in patients with HI/HA (26,27,28,29). The serum ammonia concentration of this group of patients was 85-184 µmol/l. After a confirmation of the diagnosis of HI/HA syndrome due to a *GLUD1* genetic defect, the patients were started on a low-protein diet (1.5 g/kg/day of natural protein intake). Three patients (patients 54, 55 and 56) were successfully managed by diet alone. They have had no further hypoglycaemic episodes. The other two patients were responsive to diazoxide treatment.

Of 65 patients, 40 (61.5%) achieved long-term stable glycaemic control by diazoxide alone. Octreotide was administered to three children who were not responsive to diazoxide. Among these three patients, two were unresponsive to octreotide, and one patient discontinued this drug due to severe diarrhoea. Side effects of the diazoxide treatment were observed in 32 (80%) patients. Gastrointestinal disturbances such as nausea, vomiting, severe gastrointestinal upset and poor appetite occurred in 69%. Six patients were fed through a nasogastric tube because of severe gastrointestinal reactions and their blood glucose levels were kept relatively stable. Different degrees of hypertrichosis occurred in 55% (22/40) of patients during clinical follow-up. In one case (patient 43), effective diazoxide therapy had to be stopped because of thrombocytopenic purpura.

Pancreatectomy was performed in 10 patients who were unresponsive to drugs. Nine patients were treated with subtotal pancreatectomy, and one patient underwent pancreatectomy twice. In patient 3, a second resection of the pancreas was required because of sustained hypoglycaemia (25). Histological examination of the resected pancreatic tissue confirmed diffuse disease in nine patients and atypical form in one patient. One patient (patient 7) who underwent surgery at two months of age developed diabetes mellitus at five years of age and was treated with insulin. Case 8 developed diabetes mellitus immediately after surgery and required insulin treatment. Two cases still had mild hypoglycaemia after surgery; one (patient 4) was successfully managed with regular daytime and overnight feedings and the other (patient 9) was treated with diazoxide. Only one case had symptoms of malabsorption.

Diazoxide treatment was stopped in 14 of patients (35%), between the ages of six months and four years, and no recurrence of hypoglycaemia was observed. Eight patients with subtotal resection were able to maintain normal blood glucose and HbA1c levels during the duration of follow-up. There were three diazoxide-unresponsive patients: one patient died of multiorgan failure and two patients abandoned the treatment and died of severe hypoglycemia after three to seven days at home.

## Discussion

In this study, the clinical characteristics, laboratory data and genetic features of 65 patients with CHI, the largest CHI cohort from southern China, were reported. Until now, there have been no nationwide data regarding this disorder in China, although several studies have summarized the clinical and genetic characteristics of CHI in northern and eastern China (20,21,22).

In our cohort of patients with CHI, 32.8% were noted to have disease-causing mutations: 16 (25%) patients were positive for *ABCC8* mutations; five (7.8%) were positive for *GLUD1* mutations; and 44 (67%) were negative for *GCK, GLUD1, ABCC8 *and *KCNJ11* mutations in the gene analysis. No mutations were found in the* KCNJ11 *gene in this study. As described in previous studies, most of the mutations identified have been detected in the KATP channel. The mutation detection rates of *ABCC8 *and* KCNJ11* genes reported by Kapoor et al (6) and by Snider et al (26) were 36.3% (109/300) and 69% (288/417), respectively. However, in a similar study conducted in a large group in Turkey, it was found that the mutation rate in the *ABCC8/KCNJ11* genes was 17/35 (48.6%) (30). The pick-up rate in our cohort (16/65, 25%) for *ABCC8* and* KCNJ11* gene variants is lower than those in previous reports and also differed from recent studies in China, which reported mutation rates of approximately 44% (12/27) (31) and 67.6% (25/32) (20). Accordingly, the low mutation discovery rate in our study may be due to the differences in genetic background among most of the cases from southern China. In our study, we found the deletion/insertion mutation c.3224-3226delACC ins CAGCCAGGAACTG in five patients, which may be the most common mutation leading to CHI in the southern Chinese population. These findings suggest that a geographical distribution difference may exist in the mutational spectrum of the *ABCC8* gene in the Chinese population. This mutation causes a frameshift and introduces a premature stop codon 75 codons downstream of the mutation, leading to the loss of the functional domain NBD2 (20). Five patients in this study carried compound heterozygous mutations. It was previously demonstrated that harboring compound heterozygous mutations of *ABCC8* gene was usually associated with medically unresponsive CHI (32). However, in this cohort, treatment with diazoxide was effective in three patients and one patient could be regulated with diet alone. Dekel et al (33) reported that some compound heterozygous mutations may cause milder HI which is responsive to diazoxide. Kumaran et al (34) also reported a case of transient hyperinsulinaemic hypoglycaemia due to a compound heterozygous mutation in *ABCC8*. The mechanisms responsible for this clinical variability may be related to background genetic factors and other unknown factors involved in regulating gene expression (35).

Five novel mutations were found in the *ABCC8* gene in five patients. One patient (patient 59) was a compound heterozygote with two novel deletion mutations, P.Gln273ArgfsX85 and P.Leu724del, and dietary treatment alone achieved stable glycaemic control in this patient.

Current medical management for CHI includes diazoxide combined with chlorothiazide as the first-line therapy (36). Diazoxide binds to the SUR1 subunit of the KATP channel and reduces insulin secretion by hyperpolarisation of the pancreatic β-cell plasma membrane (15). After diagnosis, a therapeutic trial with diazoxide was performed immediately. In our cohort, 40 (61.5%) CHI patients were diazoxide-responsive. Similar to our results, Kapoor et al (6) recently reported that 64% of their cohort responded to diazoxide treatment. In the cohort reported by Şıklar and Berberoğlu (19), 71% (100/141) of Turkish patients with CHI were responsive to diazoxide treatment. The recommended dosage of diazoxide is 5-15 mg/kg/day (37) and the effective dosage of diazoxide is believed to always be lower than 15 mg/kg/day. In our study, the initial dosage of diazoxide was 10 mg/kg/day and the minimal effective dosage was sought to maintain the stability of blood glucose. If the patient was unresponsive to a dosage of 10 mg/kg/day of diazoxide, further increasing the dosage did not improve the effect but rather increased the risk of serious complications. Gastrointestinal symptoms, such as vomiting, nausea and poor appetite, were common if dosages higher than 10 mg/kg/day were administered. One patient receiving effective diazoxide therapy had to stop treatment due to serious gastrointestinal symptoms and three cases required nasogastric tube feeding. To improve the effectiveness and reduce side effects, we believe that diazoxide should be used at the minimal effective dosage.

In this study, 35 of 65 patients developed mild mental retardation including four patients associated with HI/HA syndrome. Both low blood glucose and insufficient treatment increased the risk of impairment in neurodevelopment in CHI (38). HI/HA syndrome is caused by activating mutations in the* GLUD1* gene, which encodes the intramitochondrial enzyme glutamate dehydrogenase (GDH) (39). The epilepsy and developmental problems in HI/HA syndrome are thought to be a result of recurrent hypoglycaemia, chronic HA or decreased brain concentrations of the neurotransmitter GABA, due to increased GDH activity (27). The high rate of developmental delay in this study is likely to be due to the delayed diagnosis. Given that the clinical symptoms of this disease were not specific, and their HI may be less severe, more infants with CHI were misdiagnosed and not recognized until they presented with hypoglycaemic seizures, weeks to months later. The earlier determination of blood glucose and serum insulin concentrations will be helpful for diagnosis. Therefore, all neonates, infants and children should be evaluated for hypoglycaemia (40).

Dietary treatment is an important aspect of care for all patients with CHI. In our study, 18% of patients could achieve glycaemic stability with dietary treatment alone. Frequent feedings and specific diets include the provision of adequate carbohydrates to maintain normoglycaemia. The good response to dietary treatment obtained in some cases indicates that this should be the initial treatment for all CHI patients in addition to a trial of diazoxide. To increase the carbohydrate content, glucose polymer and uncooked corn starch were added to the diet of the older infants. Some infants may require a nasogastric tube for regular and frequent feedings. Patients with HI/HA syndrome require a protein-restricted diet. Feeding problems such as difficulty with sucking, swallowing, vomiting and food refusal occur in a significant proportion of children with CHI and continuous feeding through nasogastric tube or gastrostomy may be required (41).

A pancreatectomy was implemented in 10 diazoxide-unresponsive CHI patients, accounting for 15.4% of the whole cohort. During surgery, none of our patients were found to have a focal lesion. A pathological examination of pancreatic tissues revealed the diffuse form of HI in nine of 10 cases.

Our findings were similar to those reported by Bellanné-Chantelot et al (42) (58.7%; 64/109) and Li et al (43) (89.5%). In total, 40% (4/10) of patients with CHI unresponsive to diazoxide had *ABCC8* mutations. In these four patients with the diffuse form of CHI proved by histology, three cases carried a single heterozygous *ABCC8* mutation and one case carried a compound heterozygous mutation. A segregation analysis of both parents in these cases showed that the mutation was paternally inherited in three patients and biparentally inherited in one patient. CHI with a single paternally inherited heterozygous mutation in the* ABCC8* gene has been previously reported to suggest focal disease (29,37). However, Chandran et al (44) reported that heterozygous paternal mutations may also cause diffuse CHI. Paternal mutations causing diffuse disease may act via a different mechanism from that of recessive mutations (45).

### Study Limitations

Our study has limitations. Firstly, our research is a single-center study and cross-sectional design. Future multicentre studies are necessary to obtain the long-term follow-up characteristics of such patients at the national level. Secondly, other genes associated with CHI were not tested in this study. A targeted gene panel for CHI or whole-exome sequencing (WES) analysis could be applied in these patients in the future.

## Conclusion

In summary, a genetic diagnosis was made in 32% of CHI patients in this large cohort. Mutations in the *ABCC8* gene were the most common identifiable cause with a minority of variants found in the *GLUD1* gene. No mutations were identified in either *KCNJ11* or *GCK* genes. Some unique features of *ABCC8* gene mutations in southern Chinese CHI patients with more novel and hot-spot mutations were identified. Diazoxide and dietary treatment were effective in most patients. In the remaining 68% of the patients, the genetic cause of hypoglycaemia remains unknown. A targeted gene panel for CHI or WES analysis could be applied in these patients.

## Figures and Tables

**Table 1:**
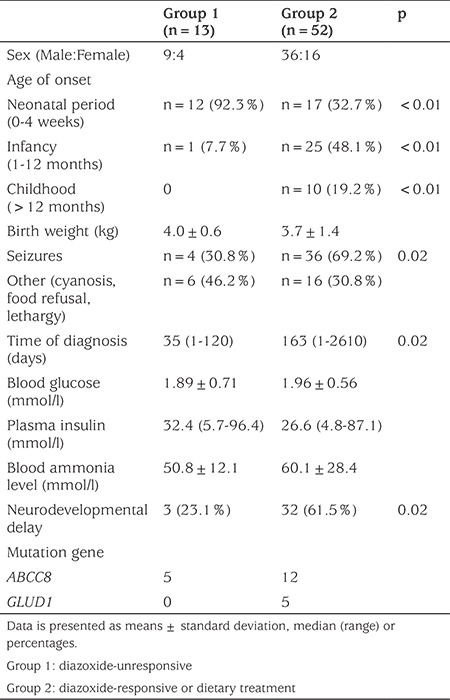
Clinical and biochemical characteristics of the congenital hyperinsulinism patients

**Table 2:**
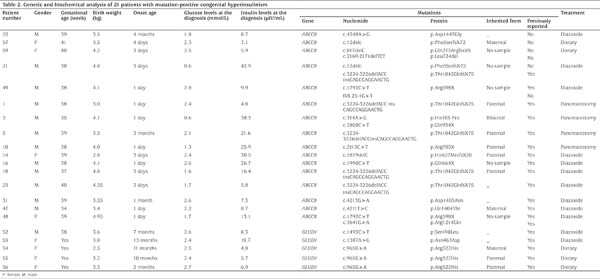
Genetic and biochemical analysis of 21 patients with mutation-positive congenital hyperinsulinism
